# The role of mirroring and mentalizing networks in mediating action intentions in autism

**DOI:** 10.1186/2040-2392-5-50

**Published:** 2014-10-14

**Authors:** Lauren E Libero, Jose O Maximo, Hrishikesh D Deshpande, Laura G Klinger, Mark R Klinger, Rajesh K Kana

**Affiliations:** Department of Psychology, University of Alabama at Birmingham, CIRC 235G, 1719 6th Ave South, Birmingham, AL 35294-0021 USA; Department of Radiology, University of Alabama at Birmingham, CIRC 235F, 1719 6th Ave South, Birmingham, AL 35294-0021 USA; Treatment and Education of Autistic and Communication related handicapped CHildren (TEACCH) Autism Program, University of North Carolina School of Medicine, Campus Box 7180, UNC-Chapel Hill, Chapel Hill, NC 27510 USA; Department of Allied Health Sciences, University of North Carolina, Bondurant Hall, CB #7120, Chapel Hill, NC 25799-7120 USA

**Keywords:** Action, Intention, Means, fMRI, Autism spectrum disorders, Mirror neuron system, Theory-of-mind

## Abstract

**Background:**

The ability to interpret agents’ intent from their actions is a vital skill in successful social interaction. However, individuals with autism spectrum disorders (ASD) have been found to have difficulty in attributing intentions to others. The present study investigated the neural mechanisms of inferring intentions from actions in individuals with ASD.

**Methods:**

Functional magnetic resonance imaging (fMRI) data were acquired from 21 high-functioning young adults with ASD and 22 typically developing (TD) control participants, while making judgments about the means (how an action is performed) and intention (why an action is performed) of a model’s actions.

**Results:**

Across both groups of participants, the middle and superior temporal cortex, extending to temporoparietal junction, and posterior cingulate cortex, responded significantly to inferring the intent of an action, while inferior parietal lobule and occipital cortices were active for judgments about the means of an action. Participants with ASD had significantly reduced activation in calcarine sulcus and significantly increased activation in left inferior frontal gyrus, compared to TD peers, while attending to the intentions of actions. Also, ASD participants had weaker functional connectivity between frontal and posterior temporal regions while processing intentions.

**Conclusions:**

These results suggest that processing actions and intentions may not be mutually exclusive, with reliance on mirroring and mentalizing mechanisms mediating action understanding. Overall, inferring information about others’ actions involves activation of the mirror neuron system and theory-of-mind regions, and this activation (and the synchrony between activated brain regions) appears altered in young adults with ASD.

## Background

As social animals, human beings are often engaged in watching and interpreting each other’s behavior, especially the ‘how’ , ‘what’ , and ‘why’ of their actions [1]. A basic understanding of the nature and means of an action helps observers infer the intention of the agent involved. Simulating others’ actions may prove critical in helping us understand the meaning of such actions and emotions, as suggested by earlier studies of the simulation theory of mindreading [2–4]. However, more recently it has been suggested that, at the neural level, the representation of one’s own and others’ actions is mediated by the mirror neuron system (MNS) through internal simulation and replication of these actions [2, 5–7]. First discovered in the macaque monkey, mirror neurons are a set of specific neurons that fire when the monkey executes a movement, as well as when it observes the same movement performed by someone else [8–12], creating a simulation circuit that allows the association of one’s own actions with the actions of others. Human neuroimaging studies have pointed to the ventral premotor cortex, especially the inferior frontal gyrus (IFG), along with the inferior parietal lobule (IPL), as the core regions of mirror neuron activity [6, 7, 9, 13–15]. Nonetheless, questions remain about the significance of the MNS and its putative involvement in making social inferences. For example, how does one understand the meaning and intent of others’ actions by simulating it? Is simulation, mediated by MNS activity, necessary and sufficient for understanding an agent’s action intention?

It is assumed that the information about a simulated action in the MNS is passed along to the core theory-of-mind (ToM - the ability to attribute mental states to others) regions for inferring an agent’s intention [16–18]. Areas found to be associated with ToM include the medial prefrontal cortex (MPFC), the posterior cingulate cortex (PCC), and the posterior superior temporal sulcus (pSTS) at the temporoparietal junction (TPJ) [19–23]. Both MNS and ToM networks are involved in understanding actions and attributing intentions, however they appear to be functionally and anatomically segregated, at least in some ways. One meta-analysis of over 200 functional magnetic resonance imaging (fMRI) activation studies showed that the MNS network (IPL and IFG) was related to processing biological motion (moving body parts) and ToM network (MPFC, PCC, and TPJ) to more abstract processing of others’ intentions, in the absence of any biological motion [24]. In addition, two cross-sectional fMRI studies [18, 25] specifically explored the how, what, and why of others’ actions in typically developing (TD) individuals. Both studies concluded that the MNS (through the IFG aspect) is active during the visual processing of others’ actions (how and what), and ToM regions (MPFC, PCC, and pSTS) are additionally recruited to process their intentions (why); thus it appears that inferring others’ intentions from their actions may be the result of two distinct functions (MNS and ToM networks).

While understanding the means of actions (how) and attributing intentions to the agent (why) may be an implicit and rather effortless task for TD individuals, several behavioral studies have found that people with autism spectrum disorders (ASD) have great difficulty in doing so [26–29]. One early study [30] suggested that an interruption in the ability to connect one’s own actions with the actions of others results in impaired social development and a deficit in ToM. Behavioral studies have indicated that individuals with ASD have a marked difficulty in connecting another’s actions to their own, particularly due to impaired imitation skills, a behavioral correlate of MNS [29, 31–33]. Since the ability to imitate may strongly rely on MNS activity [34–38], it is not surprising that a dysfunction in the MNS has been postulated as an underlying mechanism for imitation deficits seen in ASD [29, 39]. Studies have linked the MNS with other aspects of social cognition, including empathy, joint attention, self-recognition [29, 36, 40], and ToM [41]. In addition, anatomical and functional alterations of the MNS have been found to be related to social impairment and symptom severity in ASD [29, 39, 42–46] (for a review see [47]). Therefore, a dysfunctional MNS may underlie the widespread social impairments seen in ASD (see reviews by [48–50]).

Despite the findings of dysfunctional MNS, other studies have reported no differences in MNS activation in ASD participants compared to TD individuals, raising questions about a MNS dysfunction in ASD [51–53]. These critiques allude to the possibility of different neurological processes occurring at resting state in autism, triggering false detection of hypoactivation in MNS [52], the possible existence of multiple mirroring systems in autism [54, 55], and even methodological differences in fMRI studies [56]. Even the proponents of the MNS dysfunction hypothesis of ASD acknowledge that a deficit in the MNS, although able to explain some social-cognitive dysfunctions, as of yet does not explain other key symptoms of ASD, such as repetitive behaviors and restricted interests [39]. Therefore, the MNS hypothesis of ASD is a topic of intense debate in the autism literature.

The present study addresses several questions pertaining to the MNS hypothesis, the involvement of MNS and ToM networks in action understanding and mentalizing, and how these networks may be functionally different in people with ASD. Using a set of action intention stimuli adapted from de Lange *et al*. [18], we predicted a significant increase in MNS (IFG and IPL) response while attending to the means (how) of an action, whereas increased recruitment of ToM (MPFC, PCC, and TPJ) regions when attending to the intent (why) behind an action in TD individuals. Given the somewhat inconsistent literature in MNS in ASD, but consistent findings of abnormal ToM processing in ASD [57–59], we expect abnormal recruitment of the ToM network while attending to intentions, but rather typical MNS activation while attending to the means of an action in ASD participants. Based on the disrupted connectivity accounts of ASD [60–63], it is predicted that the ASD participants will exhibit altered functional connectivity (synchronization of brain activity) of the MNS and ToM networks during this task. The current study is novel in its focus on examining the differential involvement of MNS and ToM networks and their functional connectivity in autism. Findings from this study will provide new insights into understanding the role of MNS and ToM systems in action understanding and its potential alterations in ASD.

## Methods

### Participants

Functional MRI data were collected from 26 high-functioning young adults with ASD and 28 TD control participants. Five ASD and 6 TD participants with excessive head motion were excluded from the analysis. Groups were matched on age, handedness, IQ, and head motion, resulting in a final sample of 22 TD and 21 ASD participants (see Table 1). All participants were assessed on the Wechsler Abbreviated Scale of Intelligence (WASI; [64]), Edinburgh Handedness Inventory [65], Reading the Mind in the Eyes Test ([66]), the Ritvo Autism Asperger Diagnostic Scale-Revised (RAADS-R; [67]), and the Empathy Quotient (EQ; [68]). All participants were required to have a full-scale IQ of 80 or above (measured by the WASI) to be included in this study. The participants with ASD were recruited through service providers in Alabama (the University of Alabama at Birmingham (UAB) Civitan-Sparks Clinic, UA Autism Spectrum Disorders Clinic, Glenwood Foundation, Mitchell’s Place, and the Alabama Autism Society), and from a roster of individuals who have participated in previous studies from the Cognition, Brain, and Autism Lab at UAB. Participants with ASD were included if they had a diagnosis of ASD based on the Autism Diagnostic Interview (ADI-R; [69]) and the Autism Diagnostic Observation Schedule (ADOS; [70]). Diagnoses were verified through patient records retrieved through each participant’s clinician. Healthy controls were recruited through advertisements placed in the Birmingham area, and through the Introduction to Psychology course (PY 101) subject pool of the UAB Department of Psychology. Participants were excluded from the study if they reported any neurological disorders, claustrophobia, a body mass index exceeding 34, metal implants or a history of working with metal, kidney disease, diabetes, hypertension, anemia, sickle cell disease, or if they were taking psychotropic medications. Before participating in the study, study procedures were fully explained to all participants and informed consent for all aspects of the study protocol was obtained for all participants. All aspects of the study protocol and consent form were approved by the ethics committee of the UAB Institutional Review Board for Human Use (Protocol #: F070824010, Assurance #: FWA00005960).

**Table 1:** **Demographic information**

	Groups			
	TD (n =22)	ASD (n =21)	***t***value	***p***value
Gender	17 M; 5 F	17 M; 4 F	-	-
Handedness	20 R, 1 L, 1 A	16 R, 2 L, 3 A	-	-
Age (years)	24.9 (±5.2; 19-36)	25.7 (±6.4; 17-40)	0.43	0.67
Verbal IQ	114.4 (±10.3; 92-141)	112.9 (±15.0; 80-139)	0.37	0.71
Performance IQ	116.5 (±9.5; 100-133)	106.3 (±12.4; 94-138)	0.05	0.96
Full-scale IQ	117.5 (±8.8; 105-140)	116.3 (±12.8; 91-140)	0.35	0.73
RAADS-R total	45.7 (±24.1; 13-112)	115.4 (±39.5; 32-181)	3.54	<0.001
Mind in the Eyes	21 (±2.3; 17-25)	19 (±3.5; 15-25)	2.25	0.03
EQ	47.5 (±10.3; 27-74)	31.5 (±13.3; 15-65)	3.54	<0.001

Values are presented as mean (standard deviation; range). The *P* value is independent *t*-tests for differences between groups. Abbreviations: *TD* typically developing, *ASD* autism spectrum disorder, *A* ambidextrous, *F* female, *L* left, *M* male, *R* right, *IQ* intelligence quotient, *RAADS-R* Ritvo Autism Asperger Diagnostic Scale-Revised, *EQ* Empathy Quotient. EQ scores missing for one ASD participant due to incomplete questionnaire. Mind in the Eyes scores missing for one TD participant who did not complete the test.

### Stimuli and experimental design

**Figure 1:**
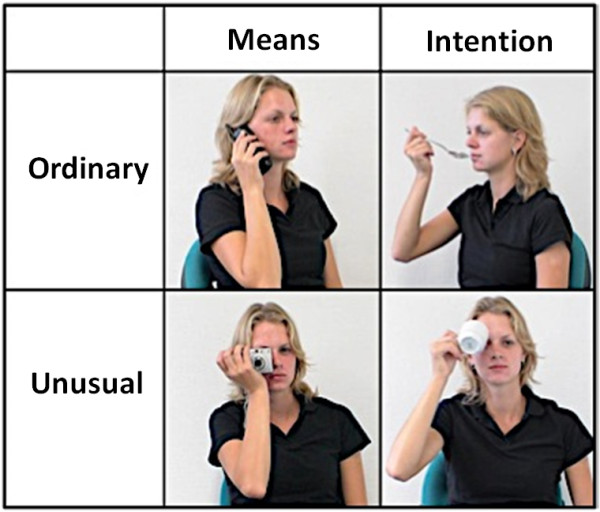
**Experimental stimuli depicting intentions, means, ordinary, and unusual conditions.**

The stimuli, adapted from de Lange *et al*. [18] included a series of static, color images of a model interacting with various common household objects (see Figure 1). The objects included were small, handheld items such as a telephone, coffee cup, spoon, or camera. The stimuli were presented in a blocked design format with two tasks (determining intentions and judging the means of actions) and a fixation condition that served as a baseline. Each image, presented for four seconds during the experiment, was unique, and presentations of the blocks were randomized and counterbalanced across participants. In the intention task, participants viewed an image and determined whether the intention behind the model’s action was ordinary or unusual (but always presented one category in a given block). In the means task, participants viewed an image and determined whether the means (or the way in which the action was carried out) of the model’s action was ordinary or unusual. Brief instructions for the tasks were presented at the beginning of each block, and participants indicated their response (ordinary or unusual) via button press. Each block consisted of four pictures with an inter-stimulus interval of one second. The experiment was comprised of 16 blocks, with eight blocks for the intention task and eight blocks for the means task. In total, 32 images per condition were included, with a total of 64 images for a complete run. Each block contained either ordinary or unusual trials, but never ordinary and unusual, to facilitate the comparison of these conditions in addition to intention and means.

Each participant practiced the experiment on a laptop computer before the scanning session. The practice included a fixation trial, as well as several unique trials (using images from the same stimuli set that were not included in the fMRI trials) representative of the intention and means conditions. While in the MRI scanner, the software Inquisit 2 (Millisecond Software, Seattle, United States) was used to visually present the stimuli. Using a laptop computer and an LCD projector, the stimuli were projected onto a screen behind the participant’s head, which were viewed using a mirror. The participants’ responses were recorded using fiber optic buttons, with unusual corresponding to the right index finger, and ordinary to the right middle finger. The participant responses provided reaction time and performance accuracy data. Behavioral data were compared between groups using a two (group) × two (condition) mixed analysis of variance (ANOVA).

### MRI data acquisition

Imaging data were acquired using a 3 T Siemens Allegra scanner (Siemens Medical Inc., Erlangen, Germany) housed at the Civitan International Research Center, UAB. Anatomical images were acquired using high resolution T1-weighted scans using a 160-slice three-dimensional magnetization-prepared rapid gradient-echo (MPRAGE) volume scan (Repetition time [TR] = 200 ms; echo time [TE] = 3.34 ms; flip angle = 12°; field of view [FOV] = 25.6 cm; matrix size = 256 × 256; slice thickness = 1 mm). Functional T2*-weighted images (T2* is a time constant describing the exponential decay of MRI signal) were obtained using a single-shot gradient-recalled echo-planar pulse sequence (TR = 1000 ms, TE = 30 ms, flip angle = 60°, FOV = 24 cm, matrix = 64 × 64. This sequence allowed rapid image acquisition and covered most of the brain (17 5-mm thick slices with a 1-mm gap in an oblique-axial orientation) in a single cycle of scanning with an in-plane resolution of 3.75 × 3.75 × 5 mm^3^. A TR of one second was used in order to elicit better temporal resolution for the functional connectivity analyses. Relatively thicker slices were used to allow for higher signal-to-noise ratio.

### Data preprocessing

Functional images were processed using a combination of Analysis of Functional NeuroImages software (NIMH Scientific and Statistical Computing Core, Bethesda, MD, USA) (AFNI, [71]) and FMRI software library 5.0 (Analysis Group, FMRIB, Oxford, UK) (FSL, [72]). Functional images were slice-time corrected, and correction for head motion was performed by registering each functional volume to the middle time point of the scan. These images were then registered to the anatomical images via FSL’s FMRIB’s Linear Image Registration Tool (FLIRT) [73, 74]. Both images were resampled (3 mm isotropic) and standardized to the atlas space of the MNI152 template via FSL’s nonlinear registration tool (FNIRT) for group comparisons, and a global full width at half maximum of 8 mm was applied.

### fMRI activation analysis

Functional images were scaled to a mean of 100 and whole-brain statistical analyses were performed on an individual basis using a standard general linear model approach via AFNI’s 3dDeconvolve function with ordinary intention, ordinary means, unusual intention, and unusual means trials as regressors of interest. Six rigid-body motion parameters acquired from head motion correction were treated as covariates. The following two orthogonal contrasts were computed to assess average brain response: intention (ordinary + unusual) versus means (ordinary + unusual), and unusual (intention + means) versus ordinary (intention + means). Areas of statistically significant activation were determined using one- and two-sample *t*-tests, and to correct for multiple comparisons, 10,000 Monte Carlo simulations were applied via AFNI’s 3dClustSim function to obtain a corrected significance level of *P* <0.05.

We further examined the relationship between fMRI blood-oxygen-level dependent (BOLD) activation driven by the tasks with neuropsychological assessment data by conducting exploratory correlational analysis between beta coefficients from the contrast intention versus means with EQ and RAADS-R total scores. The regions of interest (ROI) for this analysis were defined on the group activation map for the pooled sample (TD + ASD) using the contrast intention versus means, (peak positive and negative *t*-values) so that it best represents the ROIs for both conditions. A total of 12 ROI’s relevant to these tasks were identified. For intention: left and right middle frontal gyrus (LMFG and RMFG), left and right superior temporal sulcus (LSTS and RSTS), left and right temporoparietal junction (LTPJ and RTPJ), and posterior cingulate cortex (PCC). For means: middle cingulate (MCIN), left and right inferior parietal lobule (LIPL and RIPL), and left and right extrastriate body area (LEBA and REBA). Seeds were created using spherical binary masks (8-mm and 12-mm radius) that best captured the cluster of activation and were trimmed from white matter. Parameter estimates were then extracted from this contrast on an individual basis on each group. We assessed brain-behavior relationships separately for the TD, and ASD groups, and for both groups combined.

### Functional connectivity analysis

First, to account for signal from cerebral white matter and lateral ventricles, masks were created at the participant level using FSL’s FMRIB’s Automated Segmentation Tool (FAST) automated segmentation [75]. Masks were trimmed to avoid partial-volume effects, and an average time series for each region was extracted. Derivatives for motion parameters, white matter, and ventricular time series were computed. Following spatial smoothing, sources of noise (head motion, white matter, and lateral ventricles plus derivatives) were modeled and removed using a general linear model, and residual time series were used in subsequent functional connectivity analysis. These steps characterize this analysis as co-activation functional connectivity MRI (fcMRI) and since there is no low-pass filtering or task regression, this analysis retains task-driven effects [76]. Then, residual time series were segmented into the two experimental conditions of interest (intention and means) in order to assess connectivity between the tasks. The ROIs for this analysis were the same as the ones described in the earlier paragraph. Residual time series were extracted for each seed, and correlation coefficients were calculated across the residual time courses from other ROIs. Correlation coefficients were *z*-transformed using an inverse hyperbolic tangent function, followed by direct comparison of the *z*-transformed correlations between the TD and ASD groups using two-sample *t*-tests. Given the large number of comparisons, increasing the likelihood of type I error, a follow-up connectivity network analysis was performed by grouping the ROIs into networks based on their anatomical locations in the brain. Four networks were created for this analysis: frontal (LMFG and RMFG), temporal (LTPJ, RTPJ, LSTS, and RSTS), posterior (LIPL, RIPL, LEBA, and REBA), and medial (PCC and MCIN), and statistical analyses were performed using the same procedure as described above.

### Head motion correction

Because head motion can impact functional connectivity analyses [77, 78], the following precautions were taken. Head motion was quantified as the Euclidean distance calculated from the six rigid-body motion parameters for two consecutive time points. Any instance under 0.5 mm was considered to be excessive motion, and the time point as well as the immediately preceding and subsequent time points were censored, or scrubbed [79]. If two censored time points occurred within 10 time points of each other, all time points between them were also censored. All participants retained 80% of time points after censoring. Average head motion over each participant’s session was defined as the root mean square of displacement and did not significantly differ between groups (*t*(41) = -1.49, *P* = 0.14). For more detailed analysis of head motion, a two-way ANOVA was conducted to test the effects of group (TD and ASD) and type of motion (three translational and three rotational). The interaction of group and motion type was not significant (F-value (F)(5, 276) = 1.11, *P* = 0.41).

## Results

### Overview

The main results of this study are as follows: 1) processing intentions activated middle and superior temporal cortex along with PCC and precuneus (part of the ToM network) in both TD and ASD groups, whereas processing means activated IPL (part of the MNS network) and occipital areas; 2) processing both ordinary and unusual actions showed strong activation in both groups in frontal, middle temporal, and parietal areas; 3) group differences involved significantly reduced activation in ASD, relative to TD, in calcarine sulcus extending to PCC during intention, and increased activation in the ASD group in LIFG while processing means of an action; 4) the ASD group showed increased activation in pre- and postcentral gyrus, RIFG, and LIPL with no inverse effects found (TD > ASD) during unusual actions; 5) weaker functional connectivity was found in the ASD group between frontal and posterior temporal regions while processing action intentions; 6) brain-behavior correlations were also seen in terms of empathizing ability (positive) and autism symptom severity (negative) related to activation in PCC; and 7) a significant difference in performance accuracy, but not in reaction time, was also found between ASD and TD groups.

### Behavioral data

To assess possible group differences in performance accuracy and reaction time (RT) measured during the fMRI task, we conducted a two Group (ASD versus TD) × two condition (means versus intentions) mixed ANOVA. Group was treated as a between-subject factor and intention and means as within-subject variables. There was a significant main effect of group in performance accuracy (ASD means trials: mean (M) = 83%, standard deviation (SD) = 12%; ASD intention trials: M = 84%, SD = 12%; TD means trials: M = 92%, SD = 9%; TD intention trials: M = 89%, SD = 12%;, F(2, 41) = 5.40, *P* = 0.03). However, there was no significant main effect of condition (F(2, 41) = 0.61, *P* = 0.44), and no significant interaction between group and condition for accuracy (F(2, 41) = 1.56, *P* = 0.22). For reaction time, there was no significant main effect of group (ASD means trials: M = 1,903 ms, SD = 504; ASD intention trials: M = 1,791 ms, SD = 431; TD means trials: M = 1702 ms, SD = 436; TD intention trials: M = 1684 ms, SD = 572), F(2, 41) = 0.99, *P* = 0.33). There was also no significant main effect of condition (F(2, 41) = 1.98, *P* = 0.17), nor was there a significant interaction between group and condition for reaction time (F(2, 41) = 1.05, *P* = 0.31).

### Brain activation

#### Within-group results

**Figure 2:**
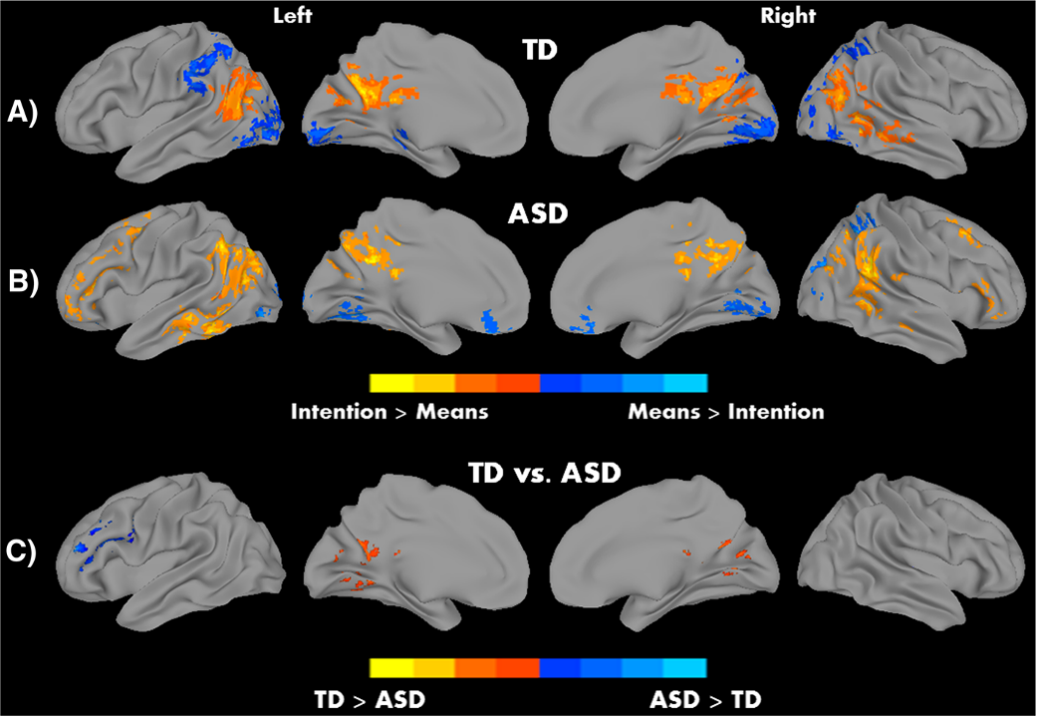
**Within-group results for A) typically developing (TD) and B) autism spectrum disorder (ASD) groups, and C) significant between-group differences for the contrast intention versus means.** Warm colors represent intention > means and cool colors means > intention for within-group results; *P* <0.05, corrected.

**Figure 3:**
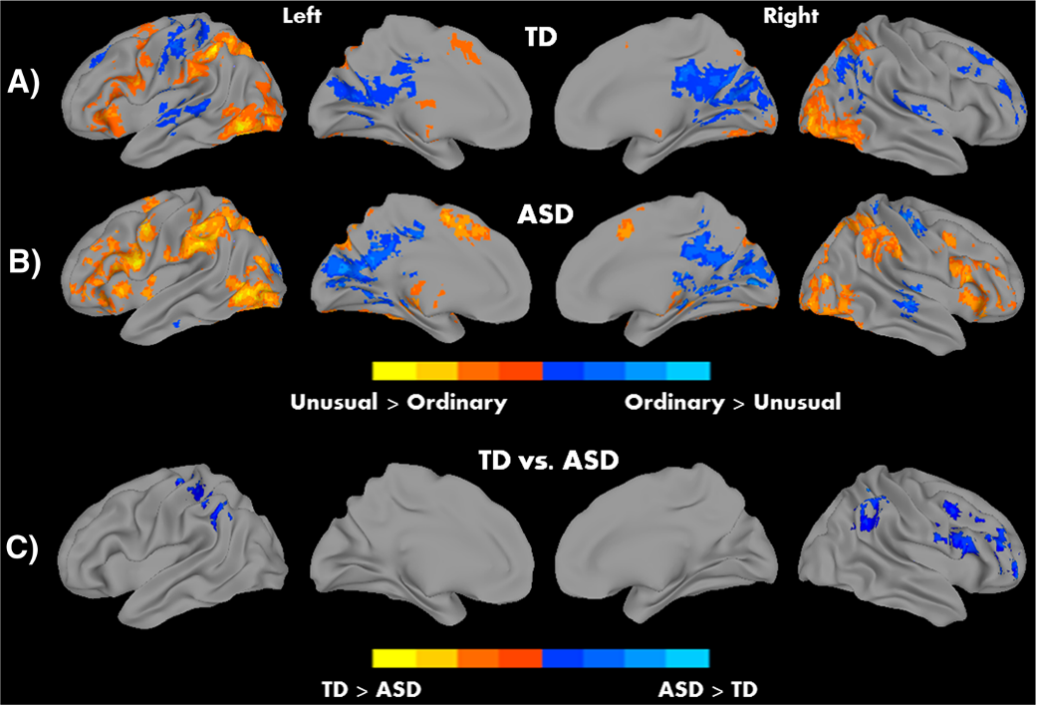
**Within-group results for A) typically developing (TD) and B) autism spectrum disorder (ASD) groups, and C) significant between-group differences for the contrast unusual versus ordinary.** Warm colors represent Unusual > Ordinary and cool colors Ordinary > Unusual; *P* <0.05, corrected.

We hypothesized the involvement of MNS and ToM networks in this task of action understanding, with differential roles in detecting the means and intention. Processing intention in contrast to means revealed robust patterns of activation in both groups in regions associated with ToM, including middle and superior temporal cortex extending to TPJ, and PCC. The means of executing an action, on the other hand, showed significant activation in bilateral IPL, and left middle/inferior occipital areas (see Figure 2A, B and Table 2). Thus, while detecting intentions recruited regions associated with the ToM network, processing means evoked activation in MNS areas like IPL. In addition, in the contrast unusual versus ordinary, activation was detected in the bilateral IPL, bilateral extrastriate body area (EBA), left IFG, and left supplementary motor area (SMA) for both groups; whereas, ordinary actions (relative to unusual actions) showed activation in postcentral gyrus and calcarine sulcus in both groups (all significant clusters at *P* <0.05, corrected; see Figure 3A, B and Table 3). There were two significant correlations between activation (for the contrast intention versus means) in the PCC with EQ, and RAADS-R total in the pooled sample (TD + ASD). The relationship between PCC and EQ was positive (correlation coefficient (*r*) = 0.33, *P* = 0.02), whereas a negative relationship was found with the RAADS-R total (*r* = -0.35, *P* = 0.01). However, these results should be interpreted with caution due to two reasons: no significant correlations were found when the groups were examined separately, and these correlations did not survive correction for multiple comparisons.

**Table 2:** **Areas of peak activation for intention versus means and means versus intention contrasts**

			Volume	Peak coordinates			Peak
Contrast and group	Region	Hemisphere	(number of voxels)	X	Y	Z	***t***
**Intention versus means**							
TD	Precuneus	Left	1,063	-6	-63	33	7.4
	Middle temporal gyrus	Right	616	39	-51	3	6.8
	Middle temporal gyrus	Left	482	-60	-63	18	5.9
ASD	Middle occipital gyrus	Left	1,056	-36	-72	36	5.2
	Posterior cingulate cortex	Left	649	-6	-51	30	5.1
	Superior temporal gyrus	Right	605	54	-45	21	5.1
	Middle frontal gyrus	Left	501	-30	3	51	5.2
	Inferior frontal gyrus	Right	102	57	21	6	3.1
	Middle frontal gyrus	Right	100	36	15	48	3.5
**Means versus intention**							
TD	Middle occipital gyrus	Left	1,564	-33	-87	-3	-5.3
	Supramarginal gyrus	Left	334	-51	-30	33	-4.0
	Thalamus	Left	143	-24	-27	-3	-4.7
ASD	Cerebellar vermis	Left	334	3	-57	-9	-3.8
	Inferior occipital gyrus	Left	253	-27	-78	-6	-4.6
	Angular gyrus	Right	127	30	-60	48	-4.6
	Rectal gyrus	Left	108	0	33	-24	-3.3
**Group differences**							
TD > ASD	Calcarine gyrus	Right	355	6	-66	15	3.8
ASD > TD	Inferior frontal gyrus	Left	178	-48	33	15	-3.4

Abbreviations: *TD* typically developing, *ASD* autism spectrum disorder.

**Table 3:** **Areas of peak activation for unusual versus ordinary and ordinary versus unusual contrasts**

			Volume	Peak coordinates			Peak
Contrast and group	Region	Hemisphere	(number of voxels)	X	Y	Z	***t***
**Unusual versus ordinary**							
TD	Inferior parietal lobule	Left	2,110	-45	-45	42	9.0
	Superior occipital gyrus	Right	1,239	30	-75	18	7.0
	Precentral gyrus	Left	1,015	-45	3	18	5.7
	Angular gyrus	Right	385	27	-63	45	7.3
	Caudate	Left	284	-9	9	6	4.5
ASD	Inferior temporal gyrus	Left	2,608	-45	-60	-6	6.7
	Postcentral gyrus	Left	2,525	-51	-3	39	6.3
	Middle occipital gyrus	Right	1,912	36	-75	12	5.9
	Inferior frontal gyrus	Right	801	33	30	-6	5.3
**Ordinary versus unusual**							
TD	Calcarine gyrus	Right	1,416	3	-60	12	-6.2
	Postcentral gyrus	Left	391	-45	-21	48	-5.2
	Middle temporal gyrus	Left	303	-45	-39	0	-5.1
	Angular gyrus	Right	216	45	-69	36	-4.1
	Superior frontal gyrus	Right	197	24	63	9	-4.0
	Middle frontal gyrus	Right	168	27	24	36	-5.7
	Superior temporal gyrus	Right	159	39	-27	0	-4.6
	Middle frontal gyrus	Left	137	-30	30	33	-4.0
ASD	Calcarine gyrus	Right	1,855	12	-78	15	-5.3
	Thalamus	Right	405	6	-27	12	-4.6
	Postcentral gyrus	Right	175	39	-27	51	-5.2
**Group differences**							
ASD > TD	Middle orbital gyrus	Right	273	48	54	-9	-4.0
	Precentral gyrus	Right	258	51	9	42	-4.9
	Postcentral gyrus	Left	246	-39	-30	60	-4.5
	Inferior parietal lobule	Right	154	45	-48	54	-4.2

Abbreviations: *TD* typically developing, *ASD* autism spectrum disorder.

#### Between-group comparison results

Between-group comparison analyses for each of the contrasts revealed significant differences between the TD and ASD groups. For the intention versus means contrast, the TD group showed increased activation in the right calcarine sulcus (extending to the PCC), whereas the ASD group showed increased activation in the left IFG. In the unusual versus ordinary contrast, the ASD group showed increased activation in middle orbital gyrus, pre- and postcentral gyrus, and right IPL. No inverse effects (TD > ASD) were found on this contrast (Figures 2C and 3C). Thus the participants with ASD showed increased recruitment of areas involved in action understanding; IFG and IPL for processing intention and unusual actions respectively.

### Functional connectivity

**Figure 4:**
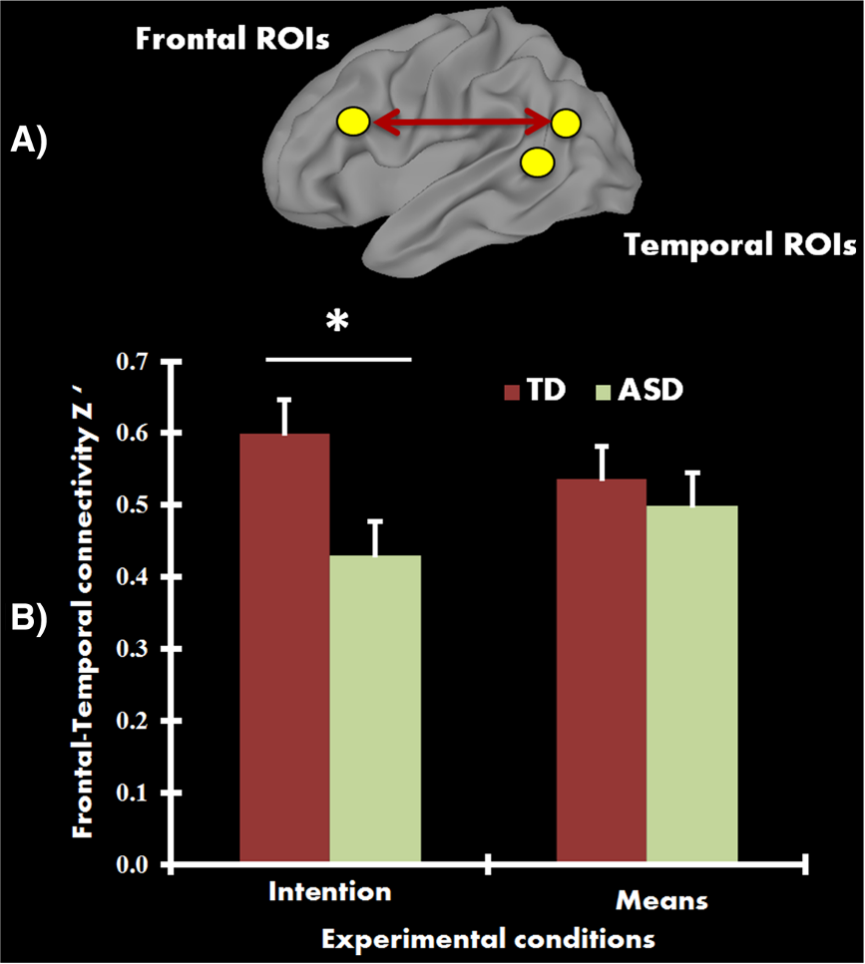
**Functional connectivity results. A)** Surface rendering showing frontal-temporal connections (regions of interest (ROIs) do not represent actual size) that revealed reduced functional connectivity in the autism spectrum disorder (ASD) group. **B)** Mean functional connectivity (*Z*’) in the frontal-temporal network for each group for intention and means conditions. Participants with ASD showed significantly reduced functional connectivity (*P* <0.01, corrected) relative to typically developing (TD) control participants in the intention condition, but not in the means condition. Error bars represent standard error of the mean.

When connectivity among pairs of ROIs were examined, the TD group showed increased functional connectivity (*r =* 0.64) compared to the ASD group (*r* = 0.53) between RSTS/RTPJ and right superior/middle frontal gyrus in the intention condition (*t*(41) = 2.12, *P* = 0.04, uncorrected). However, this effect did not survive multiple comparisons correction. Nevertheless, a follow-up connectivity network analysis revealed a statistically significant difference (corrected using the Holm-Bonferroni method) between groups, with the ASD group showing reduced frontal-temporal functional connectivity, relative to TD controls, during intention (*t*(41) = 2.90, *P* = 0.006, corrected) (see Figure 4). No significant group differences in connectivity were detected while processing the means of actions.

## Discussion

By asking participants to judge the nature of intentions and means (unusual or ordinary) of a model’s actions, this study targeted two important neural networks mediating action understanding, the MNS and the ToM, in autism. It has been a topic of debate in the field of social neuroscience as to whether these two networks are mutually exclusive [80, 81] or functionally coordinated [2, 82, 83] in the context of processing action intentions. In our study, both groups showed increased activation in middle/superior temporal cortex and PCC/precuneus, while processing intentions of actions relative to processing means of actions; regions that are considered part of the ToM network. Processing means, on the other hand, showed greater activity in inferior parietal (part of the MNS network) and occipital areas. Neurons in the IPL have been found to be critical in coding the organization of motor acts and in understanding observed actions [84, 85]. The pattern of activation seen within each group in our study suggests the use of mentalizing and mirroring mechanisms in inferring intentions and means of actions respectively. Nevertheless, it should be noted that processing intentions, relative to means, also elicited more activity in the frontal areas (MFG and IFG) in the ASD group.

This pattern was also seen in the between-group analysis, where the ASD group showed significantly more activity, relative to TD, in left IFG for processing intention. On the other hand, the ASD group showed reduced activity, for the same contrast, in the right calcarine sulcus extending to PCC. Thus, the ASD participants showed reduced activity in a core ToM network area and increased activity in a core MNS area, albeit in the left hemisphere. The left IFG peak activation was found in Brodmann area (BA) 45 and it is possible that the ASD participants depicted action intentions as pragmatic representations (the parameters that are relevant to the motor commands when executing and observing an action), which were strongly associated with the visual cue [86]. A recent meta-analysis of studies of action understanding found that bilateral BA44 and BA45 differed in activity, with the former more involved action imitation and the latter in action observation [87]. While IFG activation is attributed to MNS usually in the right hemisphere, there are several studies showing the role of LIFG in action understanding. For example, using a repetition suppression paradigm, Pobric and Hamilton showed that LIFG is necessary in making perceptual judgment about people’s actions [88]. BA45, especially in the left hemisphere, has also been associated with language production, and this increased activation may underlie communication disorders experienced by people with autism [89]. Another possible explanation is that our stimuli for identifying intentions may have targeted motor intentions, rather than abstract intentions, and ASD individuals may have processed this at the motor level with increased recruitment of the left IFG. Therefore, it is possible that ASD participants, while inferring the intent behind actions, might have relied on perceptual, motoric, and linguistic representations of actions more, as evidenced by the increased LIFG activation. It should also be noted that the TD participants in our study activated BA47, which encodes more abstract semantic representations of an action; however, this cluster did not survive multiple comparisons correction. The ASD participants did not activate this region using the same uncorrected threshold.

Most of the findings in this study are consistent with that of de Lange *et al*. [18], from where the current stimuli are adapted. Specifically, the TD group’s activation in PCC and middle temporal gyrus extending to bilateral TPJ while attending to intentions, as well as activation in bilateral occipital areas while attending to means, are consistent with this study. However, unlike the de Lange study we did not find MPFC activation at the same corrected statistical threshold at which we report other results. The MPFC has been linked primarily to self-other reflections. In the current study, the participants may have refrained from self-reflection or abstract thinking as the intentions of the model were common and evident at the motor level [90–93]. In our study, the inference about intentions may emerge from determining the model’s goal when holding a spoon (to eat) or a cup (to drink). Whereas the original study by de Lange *et al*. included images where both the how and why of an action could be unusual, our study only included unusual trials where one aspect of the action was unusual and the other was ordinary, thus making the current task relatively easier for participants. This could be another reason for the absence of MPFC activation in our task.

Previous studies have reported that prior experience, visual or motor, with an action can help in predicting the means and intentions behind that action [94–96]. Thus, the cortical mechanisms mediating a familiar action may be different from that of an unfamiliar action. In the current study, we also tested how MNS and ToM networks responded to unusual and ordinary actions and how it differed in ASD individuals. Judging unusual actions, when compared to ordinary actions, showed activation primarily in the MNS (bilateral IPL, SMA, and IFG) along with occipital regions extending to EBA in both groups. These findings are consistent with previous studies that have also reported activation in some of these regions when viewing irrational actions versus rational actions [97, 98]. In addition, occipital activation during unusual actions correspond to other studies where strong EBA response has been previously reported during incoherent actions [99]. The increased left IFG activation in the ASD group while judging means, and increased activity in the ventral premotor and IPL during unusual actions in the current study is consistent with a previous report of such an increase [46], where participants also activated MNS network during a particular task.

Reduced functional connectivity of the frontal/posterior temporal network in the ASD group while judging intentions may suggest a more isolated and less coordinated way of processing information in individuals with ASD. This weaker connectivity in ASD is in line with previous studies in adults with ASD reporting significant disruptions in frontal to posterior connectivity [58, 60–62]. The TD control participants here may be effectively engaging and coordinating frontal and temporal regions in processing actions at multiple levels (perceptual, motor, goal-oriented or intentional), ultimately inferring the intention behind actions. Such communication may not be the norm in information processing in ASD participants. While STS/TPJ and IFG belong to seemingly different, anatomically segregated networks (ToM and MNS respectively), their functional communication may be important in understanding actions at richer and more comprehensive levels. While ASD participants understand the intentions behind these actions more accurately, it is possible that their neural and cognitive route may differ from TD control participants, and may not be processing intentions at the same level as the TD control participants. It should also be noted that a group difference in connectivity was absent while processing the means of actions (*r* = 0.57 and 0.49 for TD and ASD, respectively). Efficient crosstalk between frontal and temporal regions in TD participants may underlie flexible modulation of functions (mirroring and mentalizing), which may be absent in ASD. Thus, the atypical connectivity between these two networks may be modulated by the different tasks in our study: intention, which primarily targeted ToM activity, and means, which primarily targeted MNS activity. Such a pattern has also been observed in resting-state fcMRI studies, where intrinsic BOLD signal fluctuations (as opposed to the task-driven effects in our study) are assessed [100]. Examining effective connectivity may provide more information about the transfer of information between these networks and provide more insights into the functional dynamics of this communication.

An exploratory correlational analysis revealed two significant relationships between PCC activation (intention versus means) with RAADS-R and EQ scores for all participants (ASD and TD groups combined). While these two relationships are significant, their effect size may not reveal a strong correlational effect. Nevertheless, these results are meaningful in that the activation in PCC decreased with an increase in symptom severity, and the activation in PCC increased with the ability to empathize. Finally, at the behavioral level, although a significant difference in accuracy for processing means and intentions was found when comparing the ASD young adults to their TD peers, all participants performed well above chance on the task, with overall group means for accuracy appearing to be very high (over 80% for each condition and group).

## Conclusions

In summary, we found differential activation in the TD group while processing intention and means, whereas this effect was less prominent in the ASD group. The participants with ASD showed robust MNS activity in IFG and IPL while coding intention and means of this action-understanding task. Some of these regions showed significantly increased activity in ASD participants when compared to their TD peers. Despite significant activation, ASD participants showed weaker connectivity, relative to TD controls, between frontal and temporoparietal areas while processing intention. Overall, these findings suggest a complex pattern of MNS and ToM response in individuals with ASD, with mostly intact MNS response accompanied by altered ToM activation and connectivity. Future studies should further examine the anatomical and functional roles of these systems in different aspects of action understanding and social cognition in autism.

## Abbreviations

ANOVA: Analyses of variance
ASD: Autism spectrum disorder
BOLD: Blood-oxygen-level dependent
EBA: Extrastriate body area
EQ: Empathy Quotient
fcMRI: Functional connectivity MRI
fMRI: Functional magnetic resonance imaging
IFG: Inferior frontal gyrus
IPL: Inferior parietal lobule
IQ: Intelligence quotient
MCIN: Middle cingulate
MFG: Middle frontal gyrus
MNS: Mirror neuron system
MPFC: Medial prefrontal cortex
PCC: Posterior cingulate cortex
RAADS-R: Ritvo Autism Asperger Diagnostic Scale-Revised
ROI: Region of interest
RT: Reaction time
STS: Superior temporal sulcus
TD: Typically developing
ToM: Theory of mind
TPJ: Temporoparietal junction
UAB: University of Alabama at Birmingham
WASI: Wechsler Abbreviated Scale of Intelligence.
